# Perceived public stigma and perceived public exposure by persons living with bipolar disorder: A qualitative study

**DOI:** 10.1177/00207640221093495

**Published:** 2022-05-04

**Authors:** Sophie Favre, Jean-Michel Aubry, Hélène Richard-Lepouriel

**Affiliations:** 1 Mood Disorder Unit, Psychiatric Specialties Service, Geneva University Hospital, Switzerland; 2 Department of Psychiatry, University of Geneva, Switzerland

**Keywords:** Perceived public stigma, bipolar disorder, qualitative study

## Abstract

**Background:**

Stigma impact the lives of persons living with bipolar disorder.

**Aim:**

The aim of this study was to explore how perceived public stigma is described by people living with bipolar disorder and examine the links between perceived public stigma and perceived public exposure.

**Method:**

Face-to-face in-depth interviews were conducted in a purposive sample of euthymic people living with bipolar disorder recruited in a mood disorder ambulatory unit.

**Results:**

Thematic analysis of the transcript yielded five independent themes that were related to perceived public stigma. Perceived public stigma of bipolar disorder was modeled as comprising the three elements of public stigmas (stereotype, prejudice, and discrimination), with the addition of public exposure as a core component.

**Conclusion:**

The representation of bipolar disorder in society via newspapers, films/TV series, conferences, and celebrity self-disclosures is considered to have multiple impacts. People living with bipolar disorder have also reported a perceived public stigma of bipolar disorder that has both specific features and characteristics of general mental illness.

## Introduction

Public stigma is the reaction of the general population toward people with mental illness or another stigmatized condition (Corrigan et al., 2012; Corrigan & Watson, 2002). Corrigan and Watson (2002) described the three components of public stigma as stereotypes, prejudice, and discrimination. Stereotypes are negative beliefs about mental illness (e.g. dangerousness, incompetence, character weakness), prejudices are related to agreement with stereotypes and/or emotional reactions (e.g. fear, anger), and discrimination is behavioral responses to prejudice (e.g. avoidance, withdrawal).

Perceived public stigma and awareness of public stigma may have direct or mediated consequences on psychosocial outcomes and help seeking (Link, 1987; Vogel et al., 2007). Perceived public stigma, when mediated by self-stigma, contributes to a decrease in seeking medical help (Vogel et al., 2013). Respondents with higher perceived public stigma are less likely to seek help from family and friends, while anticipated stigma is associated with seeking less formal help (i.e. that provided by a general practitioner or psychiatrist) (Pattyn et al., 2014). Moreover, perceived public stigma contributes to suicidal ideation when mediated by anticipated discrimination (Oexle et al., 2018). Perceived public stigma is also associated with greater odds of suicidal ideation, planning, and attempts (Goodwill & Zhou, 2020). Furthermore, perceived public stigma, when mediated by self-stigma, was found to lower self-esteem, raise depressive symptoms, and reduce subjective quality of life (Kao et al., 2016). Perceived public stigma is also associated with increased stigma stress in young adults at risk of psychosis or bipolar disorder (BD) in Switzerland (Rüsch et al., 2014), and stigma stress predicts reduced well-being and partly mediates the effects of perceived public stigma, shame, and self-labeling on well-being (Rüsch et al., 2014).

Research on the public stigma of BD is scarce (Kelly & Jorm, 2007), and there are inconsistent findings regarding public stigma, although there is some evidence that BD is viewed more positively than schizophrenia and less positively than depression (Ellison et al., 2013). Perceived dangerousness and fear were associated with schizophrenia, whereas labeling people with major depression had no effect on public stereotype and prejudice (Angermeyer & Matschinger, 2003). Depression also provoked significantly less negative attitudes than mania in young individuals (Wolkenstein & Meyer, 2008). In France, attitudes toward BD were less prejudicial than those toward schizophrenia (Durand-Zaleski et al., 2012). BD was primarily associated with positive beliefs and attitudes and elicited a relatively low desire for social distance in a UK population (Ellison et al., 2015). The relationship between social distance and stereotypes was partially mediated by fear; fate causal beliefs increased social distance by eliciting fear, whereas biomedical causal beliefs reduced desire for social distance by increasing compassion (Ellison et al., 2015). Moreover, misconceptions of BD were linked with negative attitudes in a Spanish population and in a population from Saudi Arabia (Alosaimi et al., 2019; Ruiz et al., 2012). Hence, the psychiatric stigma toward BD is a serious concern for people with BD and their families, with the consequences of a loss of social support, reduced functioning and quality of life, and higher symptom levels (Hawke et al., 2013). A study in Tunisia (Ouali et al., 2020) assessed stigmatization as perceived and experienced by patients with serious mental illness in Tunisia. The participants reported mostly negative perceptions or experiences, leading to apprehension about disclosure of MI. However, BD has also been primarily associated with positive beliefs and attitudes, leading to relatively low desire for social distance (Ellison et al., 2015). These results are in line with those of Johnson et al. (2016) who evidenced how creativity was attributed to BD, and its impact in stigma reduction. These different results show how public and perceived stigma of MI are shaped by structural and cultural values.

Media coverage of BD has increased over the last two decades, with TV programs, TV series, and celebrity disclosures and the promulgation by the media of the notion of BD has substantially increased awareness of the diagnosis (Angermeyer et al., 2018). Media can diffuse positive recovery stories, positive role modeling and positive portrayals that help reduce stigma (Stuart et al., 2014), and media can play a significant role in raising awareness among citizens, empowering communities to act, informing policymakers about pertinent social issues, and advocating for policy initiatives (Dinos et al., 2004). Media can thus work together with anti-stigma programs (Hildersley et al., 2020). On the other hand, while the role of media in disseminating information about mental illness is important (Ross et al., 2019), it can also be biased, sensationalized. Mistaken newspaper coverage on mental illnesses, or myths perpetuated about mental illnesses can adversely impact the public’s perception of people living with severe mental disorders (SMI) (Li et al., 2021). This negative media coverage can contribute to a harmful social environment that facilitates the rejection, discrimination, stigmatization, and marginalization of people living with a SMI (Dinos et al., 2004).

In this study the focus was on perceived public stigma, defined as awareness of public stigma. The aim of this study was to investigate the perception of public stigma of BD from the perspective of persons living with the illness, considering perceived stigma, experiences of stigma, and how the persons positioned themselves toward profiles. It also aimed to explore how perceived public stigma is described by people living with BD and examine the links between perceived public stigma and perceived public exposure. A deeper understanding of the interaction between perceived public stigma and perceived public exposure was also under consideration, given that it can provide an advanced account to improve the effectiveness of interventions. Quantitative tools cannot answer all the questions raised by the complexity of the patient experience, particularly for individuals with severe mental illness (Aubin-Ager, 2008; Carpenter & Suto, 2008). Therefore, qualitative research seemed to be the best tool for a first step, as it can fully describe phenomena in context-specific studies conducted on stigma, can inform interventions, and enable further scientific inquiry (Stutterheim & Ratcliffe, 2021).

## Methods

### Design and setting

Face-to-face in-depth interviews related to stigma were conducted in a purposive sample of euthymic people living with BD. The interviews were recorded and transcribed. Participants were recruited in a mood disorder ambulatory unit from mid-November 2018 to mid-January 2019. Adult bipolar euthymic patients were included if they were fluent in French. This study was conducted in accordance with the ethics followed at Geneva University Hospital. The study protocol was approved by the ethics committee of the canton of Geneva. Consent for the study and recording was obtained from all participants. There was no compensation.

### Data collection

The interviews were conducted by one of the investigators. Probes and reflective listening were used to elicit in-depth responses. Interviews were recorded and transcribed verbatim by a psychologist (*n* = 16) and an assistant (*n* = 6).

### Data analysis

A mixed inductive and deductive approach using a step-by-step guide (Braun & Clarke, 2006) was used for the thematic analysis of the transcripts. Two experienced raters familiar with the data read the transcripts and coded them independently. An iterative review of the themes, codes, and consensus-based discussion were used to reach agreement on the different codes and themes of perceived public stigma expressed in the transcripts during five sessions. Thematic saturation (Saunders et al., 2018) was reached after 22 interviews.

## Results

### Sample

A total of 22 euthymic participants were recruited for the study (Table 1). Two people did not wish to participate, and there was one drop out (who did not come to the appointment).

**Table 1.** Sociodemographic characteristics of the sample.

	Mean	*SD*
Age	47.1	9.140
	*n*	%
Female	15	68.2
Married or with a partner	14	63.6
Not working and/or disability pension	16	72.7

In this sample, 59.1% of people lived with BD-I and 40.9% with BD-II.

### Themes and codes

Five themes were described: 1/ Mechanism of public stigma, 2/ Features of BD, 3/ Reaction from public stigma, 4/ Public exposure, and 5/ Exposure impact. The first theme (Mechanism of public stigma) encompassed different codes: lack of information/knowledge, labeling, generalization, and banalization. In the second theme (Features of BD), different aspects of public stigma were included. Most were related to devalued representations of BD (weak, unpredictable, unproductive, dangerous, and dependent on medication). One feature had a more positive valence (creative and/or over creative). The third theme was related to reaction induced by public stigma in the general population, or among professionals such as health care professionals and employers. Public reaction encompassed codes such as fear and demeaning jokes. Professional reaction was described in mental health professionals working in general and psychiatric hospitals, or private practice. The fourth theme was public exposure, which included several codes, such as testimonials, celebrity self-disclosures, mediatization, films/TV series, and public events. The impact of public exposure was discussed in the fifth theme, which encompassed four codes (positive, double-edged, misguided, and negative). Examples of representative quotes are presented in Table 2.

**Table 2.** Representative quotes of the different themes and codes.

Themes and codes	Direct quote
Mechanism of public stigma	
Lack of information/knowledge	What I notice is that it is not well known. I find that people are not well informed, so there is a lot of confusion (female, 35–45) They don’t know and they have a rather false image of the disease. I think there is a rather false image of the disease that circulates (female, 35–45) People think that they are not very well informed about the term bipolar because they don’t necessarily know that it has to do with manic-depressive, they don’t make the link (female, 30–40)
Labeling	He’s bipolar, so we’re going to take this treatment and then we’re characterized by this DSM box (male, 30–40) Ah yes, as I was quite original, marginal, and then punk, it simply made it official (male, 55–65)
Generalization	There’s a generalization, a sort of racism. It’s like the way people used to treat gay people. Bipolar is treated the same way, so it’s hard to come out and say who you are and what you’re going through (male, 55–65)
Banalization	It’s basically mood swings and it doesn’t go further, I have the impression that it is quickly summarized for people (female, 35–45) I have the impression that this word is used a lot, well, it is used a lot, we quickly say that everything is bipolar (female, 35–45) Well, until now, when I say that I am bipolar, the person says to me, ‘Well, you are also bipolar, so and so is also bipolar’ (female, 55–65)
Features of BD (perceived stereotypes)	
Weakness	For them, you’re weak, it’s weakness. It’s not just that you’re sick, it’s that you’re weak. That’s part of the stigma (female, 45–55) Like: ‘He is fragile, we must always be careful, we will preserve him’, and uh, we will yeah, well, we will protect him (male, 30–40)
Unpredictable	Yeah, unpredictable or something like that; I think that some people have this idea (male, 30–40) We are comforted by this stigmatization and that it is much simpler, everyone says that, everyone thinks that, bipolar people are unstable and that it changes all the time and we are not going to go any further with the explanation (male, 35–45) Bipolar, you can’t confide in them, you can’t rely on them (male, 35–45)
Unproductive	Is someone who is not able to earn a living (female, 55–65) The disability insurance pays you for training and then you don’t even have to work? (female, 55–65) Well this discourse: ‘You are lazy’ (male, 30–40)
Dangerous	As I told you, in the United States, they have a prejudice that when you are bipolar or schizophrenic or PTSD, you are going to commit a crime and kill everyone, it’s really extreme, there is extreme thinking (male, 45–55) People who maybe can be dangerous sometimes (female, 40–50)
Dependent on medication	They didn’t directly tell me ‘you are addicted to this medication’. But, well, you can feel that what’s implied is ‘you wouldn’t be normal without it’ (male, 30–40)
Over-creative	They might think ‘well that’s great’ but actually it’s great because the guy is over-creative because he’s bipolar (female, 30–40) ‘Being bipolar equals being an artist, being creative’ (female, 30–40)
Reaction to public stigma (perceived prejudice and discrimination)	
Public reaction	
Fear	Very overloaded, you know, mental illnesses in general are very overloaded and scary (male, 30–40) Already the term bipolar, it’s already a term that is a bit scary (female, 30–40) But if you hear someone say, ‘I don’t want to talk to this bipolar’, you think they are really afraid of bipolar people (male, 35–45)
Jokes	I’ve also heard people in my professional environment make fun of others by saying ‘yeah, but you must be bipolar’ or things like that (male, 30–40) Once I told a colleague, who is also one of my best friends, I wanted to go to two conferences and he told me since you’re bipolar you can ask, one part of you goes to one and the other goes to the other; so there you go, it’s jokes like that, it’s things that are not denigrating, but it’s just stigmatizing not my person but bipolar in general (female, 25–35)
Professional reaction	
In general or psychiatry hospital	To treat me like an animal. They immediately picked me up and threw me on the bed and squeezed me hard and gave me an injection. That was back in the day. I don’t think people do that kind of thing anymore but it was a very brutal hospitalization. And I wasn’t violent, I hadn’t done anything. I was talking a little too fast, I had a lot of energy but I wasn’t suicidal or anything (male, 45–55) It’s not pleasant for everyone in the emergency room because they work long hours, but when you have a psychological file, you’re not received in the same way and that’s quite obvious. There are questions that you wouldn’t ask or that you would ask differently to someone . . . You wouldn’t question the word of a person who comes . . . once I arrived with my finger cut open, I was sent to the psychiatric ward first, that I didn’t understand (female, 35–45)
In the workplace	Even my boss, when he heard that I was let go (fired after a work interruption) he couldn’t understand, he was shocked. But that’s another experience I’ve had that shows once again the stigma of bipolar (male, 45–55) Well, I think they find ways to separate from this type of employee under the cover of other excuses (female, 35–45)
Public exposure	
Testimonials	Giving a testimony is to help people who are in a situation, who may have been ill or else. It’s a very good book, it’s only 90, 100 pages on her testimony plus at the end she gives recommendations on how to live with BD and on the treatments, how to treat oneself, the signs, the symptoms (male, 45–55) I read a book that was good, I think it was by a journalist or a writer who explained BD (female, 40–50). So I read a book recently by a person who was a journalist and who described a little what he had gone through, it’s true that I was very interested (male, 40–50)
Celebrities coming out	When I see that there are more and more actors or singers . . . who disclose, write a book (female, 55–65) Catherine Zeta-Jones made her coming out of bipolar (female, 35–45) There are several people who are known worldwide who made a ‘coming out’. Carrie Fisher I think. There are quite a few personalities and there are people who were diagnosed post mortem, I don’t know if that can be counted too, we are talking about Van Gogh, Churchill (female, 25–35) It shows that politicians living with BD can be strong and capable, that artists living with BD can be very creative (female, 50–60) There are a lot of cultivated people who have this disease, and there are a lot of artists, writers, singers, poets, painters who have had this disease and it shows that you can have success in life while having this disease (male, 45–55)
Mediatization	And as I was saying, the stigma is quickly established in peoples’ minds through newspapers, media, or elsewhere (male, 35–45) There was a program presented on TV (female, 40–50)
Films and TV series	There’s a movie about BD called Happiness Therapy, which is a great movie (male, 30–40) I saw a movie, I don’t know if it was Patrick Swayze or what, and you could see him getting higher and higher, his mood was rising and you could see him jumping off the walls (male, 55–65)
Public event	Because when it comes to doing Mad Pride, it’s the stigma there (female, 55–65) There are these World Bipolar Days, there are also conferences at the hospital (female, 40–50)
Exposure impact	
Positive	The fact that a lot of celebrities are starting to disclose that they’ve had bipolar disorder themselves, it’s starting to help; it’s changing the culture in America (male, 45–55) There are a lot of cultivated people who have this disease, and there are a lot of artists, writers, singers, poets, painters who have had this disease and it shows that you can have success in life while having this disease (male, 45–55)
Negative	A film I watched on BD and I thought it was too extreme, it was an American film, I thought it was too much (female, 40–50) I often find books about BD to be heavy (female, 25–35) Movies presents psychiatric hospitalization as a prison where people who are sick are treated like criminals and not like humans. So that’s another stigma of bipolar (male, 45–55)
Misguided representation	Well I didn’t really recognize BD in this movie (Happiness Therapy), but I’m not the only one who is bipolar, there are lots of different kinds of bipolarity, maybe it shows one of the aspects, but I discussed it with a friend who is also bipolar, and we had this in common, and in this film we had the impression that the person was not bipolar, that she was at the limit of normal and even if a little eccentric, it’s true (male, 30–40) I remember that I had seen the first, second season, indeed the character was described as such, I don’t remember anything very clear in relation to what I understand about BD . . . her behaviors reflected exactly that, there was a question of having to take medication or not, there was a moment when the person got carried away in her commitment and then well, she took some pretty reckless risks, but I don’t know if that really reflects anything about the disease at all (male, 40–50)
Double-edged	It was cool to be bipolar, I’ve heard that many times, well, not that anyone would tell me it’s cool to be bipolar, but there was this fashion effect, being bipolar had a side, it was the artists who were bipolar, so there was a lot of that, and then there was that again, and then if you’re bipolar and not an artist, then that’s the problem (female, 35–45) Or this personality was bipolar, so all bipolars are necessarily going to be creative, . . . they are necessarily going to be exceptions, afterward there may be expectations in relation to that, I think it’s not either . . . it’s double-edged (male, 30–40) ‘It’s double-edged, not all bipolars are going to be creative’ (male, 35–45)

### Perceived public stigma and perceived public exposure

Whereas the impact of public exposure can be understood on a continuum from a negative to a positive impact, there is a limited positive impact of increased public exposure in the society. People with BD reported pseudo-positive and negative portrayals of BD linked to a misguided representation of the disorder influenced by the exposure of BD in society via newspapers, celebrities, and films/TV series. People with BD also reported a possible evolution of public stigma by renaming manic-depressive psychosis as BD, a type of mood disorder. They also outlined how ‘bipolar’ has become a banalized, sometimes contemptuous, expression used in common language. Features of BD become more and more visible through different media, which changes the content of public stigma. Nonetheless, the stigmatizing mechanism remains as a social response to BD, pertaining the devalued condition (Table 3). Table 3 summarizes how the intensification of the media coverage, through books, films, testimonies, or public event impacts the perceived public stereotypes, without decreasing the burden of perceived prejudice and discrimination.

**Table 3.** Evolution of perceived public stigma related to the components of public stigma and the intensification of bipolar disorder public exposure.

Public stigma	Public exposure		
	+	++	+++
Stereotype	Madness	Mad genius	Unproductive
Prejudice	Fear	Banalization	Misguided knowledge
Discrimination	Marked avoidance	Microagression	Insidious avoidance/contemps

Table 3 summarizes how public exposure impacts perceived public stereotypes without decreasing the burden of perceived prejudice and discrimination. Different contents in the stereotype are temporarily related to different forms of prejudice and discrimination.

## Discussion

The aim of this qualitative research was to explore how perceived public stigma is evoked by people living with BD and describe the links between perceived public stigma and perceived public exposure. In this study, the perceived public stigma of BD was described as comprising the three components of public stigma described by Corrigan and Watson (2002) with the addition of public exposure. Corrigan and Watson (2002) showed that the public stigma of mental illness may be understood in terms of three components: stereotypes, prejudice, and discrimination. The participants in this study discussed perceived stereotypes of BD, which were either related to aspects of general mental illness, such as dangerousness, weakness, unpredictability, and unproductivity, or to specific hallmarks, such as creativity. In the literature, dangerousness has been more ascribed to schizophrenia (Ellison et al., 2015; Mandarelli et al., 2019; Vargas-Hucicochea, 2017). Stip et al. (2006) also showed that fewer people from the general population (28%) thought that individuals living with BD are violent or dangerous, compared to 54% who attributed these features to schizophrenia. Other elements of the public stigma of BD differs from the public stigma of schizophrenia and other serious mental illnesses. BD is a disease that has recently been exposed through media and it may now be considered as fashionable, especially regarding the creativity of sufferers, as mentioned by the participants in our study. An estimated 8% of people diagnosed with bipolar spectrum disorder may be highly creative (Akiskal & Akiskal, 1988). Across many decades of research, biographical studies and group studies of artists and writers have revealed an overrepresentation of cases of BD (Chen et al., 2020; Holm-Hadulla & Koutsoukou-Argyraki, 2017; Koutsantoni, 2012; Kyaga et al., 2011; Martin 2006; Wills, 2003). Research on the links between BD and creativity has highlight characteristics of persons living with BD, such as extraversion and divergent thinking (Ma, 2009). A meta-analysis focused on the relationship between creative potential, as measured by divergent thinking, and bipolar disorder (Kazcykowski et al., 2021). The two factors showed a significant yet small effect between divergent thinking and bipolar disorder. A handful of moderators were examined, which revealed a significant moderating effect for bipolar status, as either euthymic, subclinical, manic, or depressed. A recent study suggested that creativity and BD share a certain genetic vulnerability (Greenwood, 2020). These facts sustain the positive stigma of BD as creativity is positively connotated in societies. In addition to creativity, four other positive psychological traits are associated with BD: spirituality, empathy, realism, and resilience (Galvez et al., 2011). Hence, in the general population, BD is associated with positive beliefs, such as creativity and intelligence, and positive emotional reactions, such as compassion, and elicits a low desire for social distance (Ellison et al., 2015). Nevertheless, all these data on creativity should not give an overly caricatured view of BD. As some participants mentioned, one can live with BD without being creative. Moreover, negative features as weakness or unproductive were widely mentioned by the participants, far from the sometimes-overused image of creativity.

The participants also discussed perceived prejudice and discrimination. They reported demeaning jokes, for example, that were also described by other authors (Yanos, 2018). The participants of this study also reported how different contexts of discrimination, such as by health providers and employers. Both have also been described in the literature Mental health providers were reported as a source of stigma by people with mental illness (Kumar et al., 2020; Lagunes-Cordoba et al., 2021), and employees with a mental illness were perceived as having lower efficiency and self-esteem, as well as a greater vulnerability to dismissal by human resource managers (Khairallah et al., 2021).

In our study, public exposure was modeled as a core component of perceived public stigma owing to its influence on three different components of public stigma. In this sample, public exposure was discussed as related to books (autobiographies), films and TV series, conferences, testimonials, and celebrity self-disclosures. The influence of public exposure was characterized as being on a continuum from negative to positive (theme five). Media coverage of BD seems to have positive effects, in contrast to media coverage of schizophrenia. BD has been associated with literacy and creativity (Chan & Sireling, 2010) and sometimes even with a glamorous portrayal (Moncrieff, 2014). Although the impact of public exposure can be considered on a continuum from a negative to a positive impact (theme five), people with BD reported pseudo-positive and negative portrayals of BD linked to a misguided representation of the disorder influenced by exposure of BD in society. They also outlined how ‘bipolar’ had become a banalized, sometimes contemptuous, expression used in common language. The features of BD become increasingly visible through different media, which changes the content of public stigma. Nonetheless, the stigmatizing mechanism remains a social response to BD, pertaining to the devalued condition. Exposing BD in different ways is undoubtedly necessary to help build accurate knowledge of the disease. However, it seems insufficient to promote a neutral collective and well-educated representation of the disorder to move beyond separateness. Normalizing attitudes toward mental health problems can also be paradoxically perceived to enforce the concept of inevitable stigma (Paananen et al., 2020).

In our study, some participants talk about renaming of BD. The term ‘bipolar’ was introduced by Leonhard (1950) (Pignon et al., 2017) to differentiate unipolar depression from bipolar depression, and the term manic depression was changed to bipolar disorder in the third revision of the DSM (American Psychiatric Association, 1980) . The term ‘bipolar disorder’ is thought to be less emotionally loaded and be therefore less stigmatizing than the term ‘manic-depressive psychosis’ that encompasses two terms (manic and psychosis) that are emotionally loaded. Renaming manic-depressive psychosis as BD may have a destigmatizing effect, as BD is associated with less fear and social distancing (Ellison et al., 2015; Pignon et al., 2017). The term ‘psychosis’ raises fear and is associated with abnormality, whereas ‘disorder’ is less stigmatizing. Ellison et al. (2015) showed that BD is primarily associated with positive beliefs and elicits a low desire for social distancing; they also found that fear partially mediates the relationship between stereotypes and social distancing. Similarly, in Japan, the new name of schizophrenia (‘togo-sitcho-syo’, which literally means ‘integration disorder’) is less stigmatizing because it gave an impression that the condition was not irreversible but controllable (Aoki et al., 2016). This study shown also that the stigmatizing articles that linked schizophrenia and danger were increasing before renaming but started to decrease after renaming compared. However, the authors note a shift, with a number of articles on bipolar disorder and danger increased during the study period (Aoki et al., 2016). By comparison, the participants in our study also mentioned stereotypes, fear, and discrimination toward BD and pictured more negative beliefs associated with BD.

Different reviews of the effectiveness of interventions for reducing mental health-related stigma have been carried out (Morgan et al., 2018; Thornicroft et al., 2016; Waqas et al., 2020; Zhang et al., 2019). Although it is a heterogeneous field, contact interventions, education interventions, family psychoeducation programs, and anti-stigma campaigns show small-to-medium-sized reductions in stigmatizing attitudes and short-term positive changes. Short-term interventions often have a transient effect. Further research is therefore needed to investigate how to sustain the benefits and maximize effectiveness. Henderson et al. (2020) also recommended improving the understanding of pre-existing demographic differences in stigma outcomes to better understand and address the social processes that influence stigma at the individual level. Some authors suggest that anti-stigma interventions should be disorder-specific (Cassidy & Erdal, 2020; Maassen et al., 2018; Modelli et al., 2021), while other argue that discrimination against devalued minorities is similar and therefore there is no need for disease-specific approaches or context-tailored interventions (Jackson-Best & Edwards, 2018; Nyblade et al., 2019; Stangl et al., 2019; Van Brakel et al., 2019). In this study, people with BD reported specific features of the public stigma of BD compared with other mental illnesses, and most themes were related to features specific to BD. Moreover, they emphasized how the expression of BD is individual. This result advocates specific anti-stigma intervention, with a focus on the different features and intensities of BD. Additionally, the determinants of public stigma may be transversal across cultures and have cultural specificities that have to be taken into account Tan et al., 2020).

Our study has some limitations. First, given that this study is qualitative, its main aim is not to generalize the findings. Moreover, purposive sampling may limit the generalizability of the results (Etikan et al., 2016; Valerio et al., 2016). Caution is thus required when considering the external generalization of these results. Further studies on this issue are needed. Second, voluntary participation involves a bias of people being less likely to participate, such as the most marginalized or less educated. In this study, only 3 out of 25 (12%) refused to participate in the purposive sample, and we lack secondhand knowledge about this group. Third, in this sample, social media and cartoons or photos were not discussed as a means to produce or reduce stigma. The mean age of the participants was 47 years old (*SD* 9.1), which may account for the underestimation of the impact of social media on perceived public stigma destigmatization in this sample, as the most popular age group for most social platforms is between 25 and 34 (Statista, 2020).

In our study, the perceived public stigma of BD was described as comprising the three components of stereotypes, prejudice, and discrimination, with the addition of media coverage in a reciprocal relationship. The exposure of BD features in society was considered to have multiple impacts, on a continuum from negative to positive and pseudo-positive effects. The perceived public stigma of BD presents the specific features of BD and is influenced by more the general public stigma of mental illness. These results advocate specific anti-stigma interventions. Future studies could explore how public exposure could contribute to diminishing the stigma of BD.
